# Draft Genome Sequence of Staphylococcus microti DSM 22147, Isolated from the Common Vole

**DOI:** 10.1128/genomeA.00420-18

**Published:** 2018-05-17

**Authors:** XinJun Hu, Yibing Shang, Jing Guo, Huping Zhang, Yalin Liang, Jifeng Sun, Feng Yue

**Affiliations:** a The First Affiliated Hospital and College of Clinical Medicine of Henan University of Science and Technology, Luoyang, Henan, People’s Republic of China; b State Key Laboratory for Diagnosis and Treatment of Infectious Diseases, The First Affiliated Hospital, College of Medicine, Zhejiang University, Hangzhou, Zhejiang, People’s Republic of China

## Abstract

Staphylococcus microti DSM 22147 was isolated from viscera of common voles (Microtus arvalis Pallas) with generalized Brucella microti infection in the Czech Republic. To the best of our knowledge, the genome sequence of the species S. microti has not been previously studied. The complete genome sequence of strain DSM 22147 includes a genome of 2,381,859 bp (38.0% GC content) without any plasmids.

## GENOME ANNOUNCEMENT

Staphylococcus microti was first isolated in 2010 from internal organs of two common voles in South Moravia, Czech Republic (1). Another isolate was found on the skin of a small mammal (belonging to rodents or insectivores) living in northeast Poland (2). A case report describes the isolation of S. microti from milk samples collected from cows with subclinical (in most cases) and clinical mastitis (3). To date, very little is known about the occurrence and pathogenic potential of S. microti. It has been difficult so far to fully assess the real pathological and epidemiological significance of the microorganism. Here, we report the draft genome of S. microti strain DSM22147, the first genome of the species S. microti to be sequenced.

Strain DSM 22147 was grown aerobically on Columbia blood agar base at 37°C for 24 h. Genomic DNA was extracted using the DNeasy blood and tissue kit (Qiagen, Germany). The quantity of DNA was measured with a Qubit fluorometer. Then, 10 μg of DNA was sent to Zhejiang University.

One DNA library was generated (422-bp insert size, with the Illumina adapter at both ends), and then sequencing was performed by using an Illumina HiSeq 2000 genomic sequencer, with a 2 × 100 paired-end sequencing strategy. Clean filtered reads were assembled into scaffolds using Velvet version 1.2.07 (4), and then we used a PAGIT flow (5) to prolong the initial contigs and correct sequencing errors. Predicted genes were identified using Glimmer version 3.0 (6). tRNAscan-SE version 1.21 (7) was used to find tRNA genes, whereas ribosomal RNAs were found by using RNAmmer version 1.2 (8). To annotate predicted genes, we used HMMER version 3.0 (9). The KAAS server (10) was used to assign translated amino acids into KEGG Orthology with the single-directional best hit (SBH) method. Translated genes were aligned with the COG database using NCBI BLASTP.

The draft genome sequence of strain DSM 22147 revealed a genome size of 2,381,859 bp and a G+C content of 38.0% (186 scaffolds with an *N*_50_ of 77,222 bp). These scaffolds contain 2,175 coding sequences (CDSs), 43 tRNAs, and 25 rRNAs. A total of 2,175 protein-coding genes were assigned as putative functional or hypothetical proteins, and 2,143 genes were categorized into cluster of orthologous groups (COG) functional groups.

To the best of our knowledge, the genome of S. microti has not been previously reported. The complete genome sequence will help to advance our understanding of the biodiversity of Staphylococcus phages. Also, the current study will provide valuable information for further genomic study of phages.

### Accession number(s).

This whole-genome shotgun project has been deposited at DDBJ/EMBL/GenBank under the accession no. JXWY00000000.
